# [Retracted] Inhibition of 5‑lipoxygenase triggers apoptosis in pancreatic cancer cells

**DOI:** 10.3892/or.2024.8853

**Published:** 2024-12-09

**Authors:** Guo-Xiong Zhou, Xiao-Ling Ding, Sheng-Bao Wu, Hai-Feng Zhang, Wei Cao, Li-Shuai Qu, Hong Zhang

Oncol Rep 33: 661–668, 2015; DOI: 10.3892/or.2014.3650

Following the publication of the above paper, a concerned reader drew to the Editor's attention that Fig. 3 on p. 664, showing TUNEL assay data relating to apoptosis of the cell line under investigation in this paper, contained apparent anomalies, including repeated patternings of certain cells both within and between the data panels, such that it would have been difficult to have attributed these anomalies to coincidence.

After having conducted an independent investigation in the Editorial Office, the Editor of *Oncology Reports* has determined that the above paper should be retracted from the Journal on account of a lack of confidence concerning the originality and the authenticity of the data. The authors were asked for an explanation to account for these concerns, but the Editorial Office did not receive a satisfactory reply. The Editor regrets any inconvenience that has been caused to the readership of the Journal.

